# Research and Progress on the Mechanism of Iron Transfer and Accumulation in Rice Grains

**DOI:** 10.3390/plants10122610

**Published:** 2021-11-28

**Authors:** Qian Wang, Mengjie Chen, Qianyi Hao, Hanlai Zeng, Ying He

**Affiliations:** MOA Key Laboratory of Crop Ecophysiology and Farming System in the Middle Reaches of the Yangtze River, College of Plant Science and Technology, Huazhong Agricultural University, Wuhan 430070, China; SamWang@webmail.hzau.edu.cn (Q.W.); chenmj@webmail.hzau.edu.cn (M.C.); haoqy@webmail.hzau.edu.cn (Q.H.); zenghl@mail.hzau.edu.cn (H.Z.)

**Keywords:** rice grain, iron uptake, transport and accumulate, nutritional strengthen, genome editing techniques

## Abstract

Iron (Fe) is one of the most important micronutrients for organisms. Currently, Fe deficiency is a growing nutritional problem and is becoming a serious threat to human health worldwide. A method that could help alleviate this “hidden hunger” is increasing the bioavailable Fe concentrations in edible tissues of major food crops. Therefore, understanding the molecular mechanisms of Fe accumulation in different crop tissues will help to develop crops with higher Fe nutritional values. Biofortification significantly increases the concentration of Fe in crops. This paper considers the important food crop of rice (*Oryza sativa* L.) as an example and highlights recent research advances on the molecular mechanisms of Fe uptake and allogeneic uptake in different tissues of rice. In addition, different approaches to the biofortification of Fe nutrition in rice and their outcomes are described and discussed. To address the problems that occur during the development and application of improving nutritional Fe in rice, technical strategies and long-term solutions are also proposed as a reference for the future improvement of staple food nutrition with micronutrients.

## 1. Introduction

Micronutrients play a vital role in activities of life and human health. The lack of micronutrients, also called “hidden hunger”, is an increasingly serious and global health problem that humans face. As one of the important micronutrients, iron (Fe) deficiency is also prevalent in worldwide populations. Anaemia affects one-third of the world’s population with iron deficiency anaemia (IDA) being the top cause. IDA is highly prevalent in preschool children (<5 years), women of reproductive age and pregnant women, with prevalence rates reaching up to 39.8%, 29.6% and 36.5%, respectively [1]. Anaemia is more largely caused by the difference in the degree of social and economic development of the countries, but the specific cause is the insufficient daily dietary intake of nutritional trace elements. Prevention strategies and mitigation measures of adding Fe supplements and optimizing dietary structure have been utilized around the world in the last two decades but have not significantly improved Fe nutrition levels for the global population [2]. Improving the endogenous Fe concentration of staple food should be a main goal for developing countries as a sustainable strategy because dietary components as sources of iron have only resulted in limited improvement [3].

Rice (*Oryza sativa* L.) is an important cereal crop and a staple energy source for half the global population [4]. Based on the extensive consumption and wide variability of the grain, enhancing its endogenous Fe concentration or optimizing the Fe storage form, should be an effective way to increase not only the nutritional Fe supply of the diet but also its biological utilization efficiency. These methods might be basic and feasible ways to alleviate global Fe deficiency, particularly in developing countries [3]. Hence, this study investigates differences in Fe concentrations and the food safety range of different genotype rice varieties, describes the mechanisms of Fe uptake, distribution, and accumulation from soil to seed, summarizes the progress of biological strengthening for rice grain Fe in the past two decades, and offers a new direction to focus.

## 2. Physiological Function of Fe and Its Nutritional Requirements in Organisms

### 2.1. Physiological Function of Fe in Organisms

Fe is one of the essential elements for the activities of life. Biological endogenous Fe often exists in multiple redox forms and is widely involved in multiple physiological processes, including cytochrome and ferritin formations, electron transfer processes, and redox reactions [5]. During plant growth, Fe deficiency usually reduces leaf chlorophyll concentration and photosynthesis, leading to the leaves losing their green colour, yellowing, or gaining discolouration. In severe cases, the root epidermis can become necrotic, which can lead to stunted growth or even death. Although the Earth’s crust is relatively rich in Fe (Fe composes approximately 5% of the crust), approximately 25~40% of the world’s arable land is still severely deficient in effective Fe. This is especially apparent in alkaline or neutral arable land in which Fe deficiency is particularly pronounced and Fe is present in an oxidized state, which is not conducive to the use and uptake by plants. When crops are grown under such conditions, their normal growth and development are restricted, resulting in reduced yields and lower quality, which causes serious losses to agricultural production [5].

For animals and humans, Fe is a functional component of haemoglobin, myoglobin, and oxidoreductase, which is involved in numerous physiological processes to ensure the normal activities of life can occur, such as oxygen transport, exchange, and respiration. IDA is caused by a lack of Fe intake in humans, resulting in cognitive and immunity decreases in special populations and an increased risk of maternal mortality during perinatal periods [6]. Three effective strategies of mineral supplements, food fortification, and nutrient enrichment are proposed to combat Fe deficiency. The implementation of the first two strategies is complex and not very affordable in developing and less developed countries but the third strategy is expected to be an effective and lasting approach [7]. Cereal crops are the main source of Fe intake for humans and animals, and consuming more Fe-rich food crops can supply more Fe to alleviate the problem of Fe deficiency. Especially in Asian countries where 90% of the world’s rice is produced and consumed [8]. Despite the high level of Fe in cultivated soil or rice grains, the effectiveness and rate of biological utilization of Fe from rice is low. On the one hand, rice is traditionally cooked after milling and polishing, reducing the nutritional value because of the removal of the Fe-rich bran and embryo, with only the endosperm remaining, On the other hand, the main Fe-chelation form of rice is not well-absorbed by human intestines [9]. Therefore, the Fe level in food crops and rice grains in particular is directly related to the crop nutritional level and health status of humans. Increasing the effectiveness and available Fe in rice grains is a key strategy for developing countries to relieve Fe deficiency in their populations.

### 2.2. Range Variation of Fe Concentration in Rice Grains and Demand of Fe Concentration for Human

Grain Fe concentration is greatly affected by different rice genotypes. The grain Fe concentration of brown rice was investigated from 113 major cultivated rice varieties in China and the results revealed a variation range of 12.16~53.62 mg kg^−1^ and a mean value of 24.59 mg kg^−1^ [10]. In polished rice grain, the Fe concentrations in 598 rice resources and breeding materials worldwide were only 0.86~11.34 mg kg^−^^1^ [11]. Lu et al. (2013) found brown rice includes bran, endosperm, and embryos, which have significant tissue-specific differences in the micronutrient levels, such as the Fe concentration. Rice bran usually contains large amounts of micronutrients with Fe levels up to 86~430 mg kg^−1^. However, the Fe concentrations of brown and polished rice differ from 2~52 mg kg^−1^ and 2~28 mg kg^−1^, respectively [12].

For humans, the average daily Fe requirements are different based on individual ages, genders, and weights, with a range of 8~18 mg day^−1^. Pregnant women in particular have a Fe requirement of over 30 mg day^−1^ [13]. Moreover, excessive micronutrient intake can be detrimental to human health; therefore, the proposed maximum limit of Fe in grains is 50 mg kg^−^^1^ [14]. Considering the daily consumption of rice, researchers have proposed that the basic standard of the nutritional Fe level in rice grain should reach 15 mg kg^−1^ [15]. There is still a gap between the human nutritional requirements and the improvement of crops or staple food nutrition despite the great progress that has occurred in crop nutritional biological reinforcement research [2]. For the main planted rice varieties, the endosperm Fe level and utilization should be emphasized, which has potential for nutritional improvement.

## 3. Fe Absorption and Transport Mechanism in Rice Plants

### 3.1. Absorption Mechanism of Fe into Rice Roots

To adapt to different soil or environmental conditions, plants have developed two different strategies to secure Fe in roots, namely, the reducing strategy (I) and the chelating strategy (II). Non-grass plants adapt to strategy I, in which the soil pH is directly reduced and the available Fe^3+^ is increased by protons flowing from the root cell to the rhizosphere. Then, ferric chelate reductase (FRO) on the root surface catalyzes the reduction of surface Fe^3+^ into Fe^2+^. Finally, Fe^2+^ is directly transported to the root cell by iron-regulated transporter 1 or iron-regulated transporter 2 (IRT1/IRT2). However, in the Gramineae adaptation strategy II, the root cells exocytose the pytosiderophore (PS), which chelates with Fe^3+^ in the soil to form Fe-PS. Then, Fe-PS is transported to the root via the yellow stripe-like (YL/YSL) transporter family and enters the root cell [16,17].

**Figure 1:**
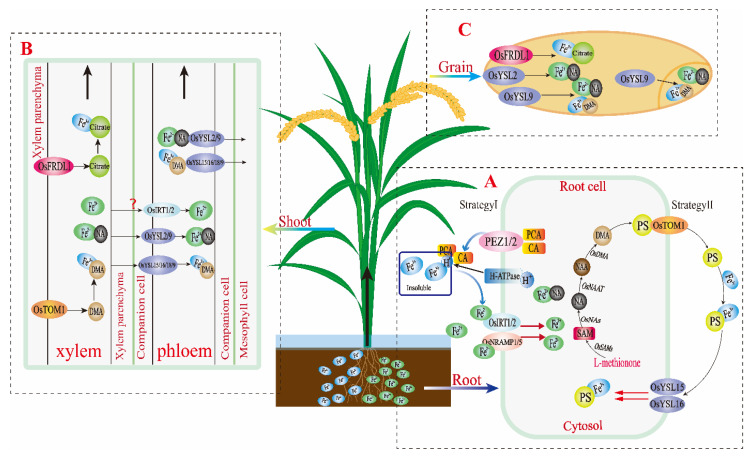
Schematic diagram for Fe uptake mechanisms in roots through strategy I, strategy II, Fe-transport from roots to shoots and grains in rice plant. Absorption mechanism of Fe into rice roots (**A**). Fe-transport mechanism from root to shoot (**B**) and grain (**C**) in rice plants.

The above two absorption mechanisms have been demonstrated in rice. However, it has been reported that FRO has very low activity on the surface of rice roots. Because of rice roots exposure in anaerobic conditions, Fe^2+^ is abundant and can be absorbed directly without activation of FRO. The mechanism by which soil Fe^2+^ is directly absorbed is partially mediated by the Fe transporter OsIRT1 and OsIRT2 that is anchored in the rice root cell membrane [18,19]. In addition, the natural resistance-associated macrophage protein (NRAMP) family members OsNRAMP1 and OsNRAMP5 positively regulate rhizosphere Fe^2+^ absorption in rice (Figure 1A) [20,21]. Furthermore, the root phenolic outflow allows the occurrence of insoluble Fe in solution from the soil. For example, rice root efflux of protocatechuic acid (PCA) into the soil can increase Fe availability to assist root Fe absorption using strategy I (Figure 1A) [22,23].

Current mechanistic studies have indicated that the most important chelator for Fe is mugineic acid (MA) in rice. The MA family is synthesized through a conserved pathway that was originally from S-adenosine methionine, in which the three synthases are nicotianamine synthase (NAS), nicotianamine aminotransferase (NAAT) and deoxymugineic acid synthase (DMAS). DMAS mediates deoxymugineic acid (DMA) synthesis and is further hydroxylated to MA (Figure 1A) [24,25,26]. In rice roots, only DMA is secreted into the rhizosphere to bind with rhizosphere Fe^3+^ and form the Fe(III)-DMA complex, which is then transported via MAs efflux transporter 1 (OsTOM1). In addition, the Fe(III)-DMA form can be transported to root tissue with the assistance of YSL family members OsYSL15 and OsYSL16 [27,28]. Nicotianamine (NA), an intermediate product of MA synthesis, also combines with Fe^2+^ to form Fe(II)-NA. Subsequently, the Fe-chelated form is transferred to other desired Fe tissues and is involved in several activities, such as photosynthetic and electron transport chain activity in leaves, is stored reproductive organs, and participates in seed development (Figure 1A) [29].

### 3.2. Fe Transport Mechanism in Rice Plants

The upward movement of Fe ions from root cytosol to young branches, leaves, and other tissues through xylem and phloem, which relies mainly on the action of root pressure, transpiration pull, and transporter proteins. Usually, DMA and NA are required for chelated Fe transport in rice, which thereby avoids excessive Fe ions precipitation, reactive oxygen species caused by Fenton reaction to damage cells [30,31]. In addition, some other chelating agents are involved in Fe transport between rice tissues, such as citrate NA and DMA. The form of Fe(III)-citrate is mostly actively transported through loading and the multidrug and toxin efflux family (MATE) and protein ferric reductase defective like 1 (OsFRDL1) (Figure 1B,C) [32,33]. In particular, in rice phloem, the nonprotein peptides of NA and DMA assist in Fe active transport with Fe(II)-NA and Fe(II)-DMA chelation via the YSL specific transporters between tissues. Whether the IRT transporter is involved in the transport of Fe^2+^ is still controversial and needs further study (Figure 1B).

The YSL family consists of 18 members in rice, of which OsYSL15 and OsYSL16 have been identified to participate in the rhizosphere absorption of Fe and phloem Fe(III)-DMA transport [28,34]. OsYSL18 is involved in Fe(III)-DMA transport in reproductive organs, crown root epidermis, the phloem of leaf sheath and accompanying cells (Figure 1B) [35]. Notably, OsYSL2 is specifically involved in Fe(II)-NA transport into rice seeds [30,36]. Recently, it was found that both Fe(II)-NA and Fe(III)-DMA can be transported into rice grains via OsYSL9 and are involved in grain Fe distribution and transport, especially from endosperms to embryos (Figure 1C) [37]. Studies of chelators and transporters enrich our understanding of the mechanism of Fe accumulation and homeostasis in rice plants.

At present, there is still a gap in the understanding of the Fe absorption and transport mechanisms in rice, especially their transport mechanisms such as the possible chelator types, individual Fe transport quantities and contributions, and their tissue-specific functions in different transport mechanisms. Therefore, the molecular mechanisms of Fe transport, accumulation, and redistribution within rice tissues need to be studied further.

### 3.3. Accumulation Form and Distribution Characteristics of Fe in Rice Grain

The development of technology allows for Fe distribution to be more clearly understood in plants. Perls Prussian blue stain has revealed that Fe was unevenly distributed in rice grains and most of the Fe was in the embryo, the secondary Fe was in the pericarp and aleurone layers and the least amount of Fe was in the endosperm. The Fe distribution was also disproportionate, particularly in embryos, with the strongest distribution in the shield, the middle distribution in the germ sheath and the weakest distribution in the root sheath [38]. The refined distribution of grain minerals has been reported through synchrotron based on X-ray and fluorescence techniques, and most of the grain Fe was concentrated in the rice husk and bran cell layers [12,39], characterizing the Fe distribution in rice grain using inductively coupled plasma mass spectrometry, which was bran > shell > whole-grain > brown rice > polished rice [40].

Fe homeostasis is tightly regulated throughout plant growth. As mentioned above, Fe is mostly absorbed and transported by binding to various chelators for plants. Simultaneously, these chelators, including organic acids (phytic acid, citrate and malate), amino acids (histidine), inorganic ions (SO_4_^2−^ and HCO_3_^−^), short peptides (DMA and NA), and ferritin (FER), also chelate endogenous Fe for accumulation and storage in plant tissues. Among these chelators, the main chelators in rice are NA, DMA, FER, and citrate [41,42]. By phosphorus (P) and Fe tracers, studies have shown that grain Fe is mostly stored in the protein storage vacuoles (PSVs) of rice grains by binding to phytic acid (PA) [43]. Moreover, it is believed that FER is the main Fe storage protein not only in plants but also in animals, which can store up to 4500 Fe atoms and can also be released with the bioavailable form of Fe^3+^ [44]. In general, these FERs are located in nutrient plant organs, such as leaf vacuoles and chloroplasts. There are two *FER* genes, *OsFER1* and *OsFER2*, in the genome of rice, and neither of them are detected in normal developing or mature seeds. Rice seed OsFER can be induced by exogenous Fe during its germination under dark conditions, suggesting that the main role of OsFER in seed is to protect tissue against free ferritin-mediated oxidative stress instead of Fe storage [45]. When *OsFER* is endosperm-specifically expressed through the promoter regulation of globulin or glutelin, the rice endosperm Fe concentration is still increased by 2.0~3.7-fold [46]. Notably, Kyriacou et al. (2014) performed a comparative analysis of grain Fe accumulation in wild-type and reinforced rice and found that numerous Fe(II)-NA and Fe(III)-DMA accumulated in the aleurone layer instead of the Fe-PA form. At present, little is known about the specific form, contribution rate, chelate type, and composition of Fe location and accumulation, especially in rice grains [47]. Therefore, it is necessary to further study and clarify the mechanism of Fe enrichment in rice and provide a theoretical basis for a new molecular strategy to enhance the amount of bioavailable Fe in staple food.

## 4. Interrelationship and Balance between Fe and Other Elements in Rice

### 4.1. Interrelationship and Balance between Fe and Other Metal Elements in Rice

A balance between endogenous metal elements is essential for maintaining normal metabolic activities in plants. In particular, various metal elements must be strictly supervised and maintained to ensure their optimal concentrations, maintain their physiological functions and avoid oxidative damage that results from their excessive accumulation. It has been reported that different divalent metal ions could compete and interact, which affected their forms or contents in plants [48,49]. For example, zinc (Zn), manganese (Mn), and cadmium (Cd) generally share an absorption mechanism with Fe, which commonly formats and regulates the homeostasis of endogenous metal elements in plants.

As mentioned above for the two mechanisms of Fe absorption in the soil-source Fe^2+^ and Fe^3+^, these processes and homeostasis are also affected by other metal cations, such as Zn^2+^, Mn^2+^, and Cd^2+^ [50]. Metal-transport membrane systems can transport all kinds of metal cations, which may be a key aspect of their competition. For example, IRT1/IRT2 belongs to the zinc-/iron-regulated transporter-like protein (ZIP) family, mediating multiple metal transport of Fe^2+^, Zn^2+^, Mn^2+^ and Cd^2+^ [51]. In rice, OsIRT1 and OsIRT2 are associated with the absorption of bivalent metal ions, and the tissue Fe contents can increase via OsIRT1 overexpressing plants, but also promote Zn or Cd accumulation in roots and aboveground tissues [52]. Furthermore, both the OsZIP1-4 of ZIP family and OsHMA2 of Heavy Metal Atpase (HMA) family can transport bivalent Fe^2+^ and Zn^2+^; OsNRAMP1 and OsNRAMP5 of the NRAMP family can also transport different metal ions of the same valence state, such as Fe^2+^, Mn^2+^ and Cd^2+^ [53,54]. In this pathway, important chelates, DMA and NA, are involved in the transport of other metal ions such as Zn2+, Mn2+ and Cu2+ in addition to Fe^3+^ and Fe^2+^ [55]. The overexpression of homologous or heterologous NAS in rice plant significantly increases not only the Fe contents but also the Zn, Cu, and Mn contents in transgenic plants [9,56,57,58,59,60,61]. Accordingly, there is competition or synergy between the absorption and transport of Fe and other metal elements, including Zn, Mn, Cu and Cd.

It seems possible that enhancing nutritional Fe concentration is accompanied by a reduction in certain heavy metal levels in cereal crops. Studies have shown that a method of applying Fe fertilizer can improve crop Fe concentration and effectively reduce the levels and toxicity of Cd to some extent. This effect can be explained by the competition of Fe^2+^ and Cd^2+^ for the same binding site and transport system on root surface cells, leading to a trade-off between the absorption of the two metals [62,63,64]. On the other hand, Fe^2+^ is an important cofactor of antioxidant enzymes, and plant oxidation resistance significantly improves with the increase and improvement in antioxidant enzyme upregulation in plants, forming a protective barrier against Cd toxicity [65]. In rice production, the application of Fe fertilizer also reduces lead (Pb) induced cytotoxicity, which is similarly due to competition between Fe and Pb for transport proteins and long-distance transport-associated chelators, as mentioned above [65,66].

Many studies on metal homeostasis have been carried out, but there are still gaps in our understanding of the overlapping mechanism of metal absorption and transport, physiological effects and coordination between metals and unknown molecules in their interaction network. Knowledge on the mechanisms of metal absorption, transport and homeostasis will intensify research efforts to better understand mechanical complexity and to provide a theoretical basis to improve crop nutrition reinforcement strategies.

### 4.2. Interaction between Fe and S and P Elements

Sulfur (S), one of the essential nutrients for plant growth processes, is a resistance factor against biotic and abiotic stresses. The homeostasis of various elements is continuously focused on in plants, especially for the interactions and linkages between Fe and S [67]. To respond to exogenous S nutrient supply, the Fe activation and translocation in plant can be up-regulated from plastid to roots or aboveground tissues, mainly due to the S-induced synthesis of substances, such as cysteine (Cys), glutathione (GSH) and phytochelatin (PC). The substances also provide the raw materials and substrates for the Fe chelations and transports synthesis. NA, a carrier molecule mentioned above, is one transport-related molecule for Fe chelation derived for Cys [67,68]. In rice, the previous study has been shown that an appropriate supply or application of the S element could achieve an enhancement of Fe uptake and accumulation with the synergistic effects [69]. The molecular mechanism of the interaction between S and Fe is still poorly understood, which needs focus on in the further.

Phosphorus, an essential macronutrient for crop growth, is widely known with the phosphate (Pi) form for uptake in plants. Fe can interact with Pi in the soil, growing medium, plant root surface. In the antagonistic manner of Pi and Fe interact, the Pi deficiency has been increased the Fe concentration in rice seedling shoots, while not affected in its roots [70,71,72]. It has been shown that the Fe intensification is attributed to the response of the Fe-responsive genes induced to the Pi deficiency under the P-deficient conditions for plants [73]. In other words, the Fe deficiency also contributed to the Pi accumulation in plants. The results of experiments with Pi- and Fe-treated Arabidopsis seedlings suggest that the effectiveness of Fe affects the response of lateral roots to Pi deficiency [71,74].

Recently, studies on the molecular basis of the interaction between Pi and Fe in plants have made outstanding progress. Under Pi deficiency in plants, the core transcription factors of the phosphate starvation response, PHOSPHATE RESPONSE 1 (PHR1) and PHR1-LIKE 1 (PHL1), control the expression of the Pi transporter proteins, PHOSPHATE TRANSPORTER1 (PHT1) and PHOSPHATE1 (PHO1). Moreover, PHR1 and PHL1 act as the positive regulators of the transcription of Fe transporters *FER1*, *NAS3*, and *YLS8* responsible for Fe homeostasis in plants [75,76]. On the other hand, some other transcription factors involved in the interaction mechanism between Pi and Fe, including LOW PHOSPHATE ROOT1 (LPR1), PHOSPHATE DEFICIENCY RESPONSE2 (PDR2), as well as the SENSITIVE TO PROTON RHIZOTOX-ICITY1 (STOP1) and its target *ALUMINIUM ACTIVATED MALATE TRANSPORTER 1* (*ALMT1*). For example, the Pi deficiency had induced a STOP1-modulated signaling cascade (PDR2-LPR1-STOP1-ALMT1) to promote the Fe accumulation in roots [77,78]. In addition, the Pi-dynamics changes had also induced Mitogen-activated protein kinase (MAPK)-mediated Pi sensing, signaling and responses in plants, whereas the Fe deficiency had reduced a MAPK6 activity to repress PHT1 family genes downstream of the WRKY75 transcription factor, suggesting that a mechanism in coordinating Pi and Fe nutrient responses [79]. In rice, although root growth is either enhanced or unaffected by low Pi, at least one component of the signaling pathway controlling local Pi-deficiency responses (LPR ferroxidase) has been shown to affect Fe accumulation and Pi translocation. Additionally, the potential involvement of PHR1 in the Pi-Fe signaling crosstalk has been proposed [80]. A recent new study shows that the notable Hemerythrin motif-containing Interesting New Gene- and Zinc-finger proteins (HRZs) and PHRs, forming a mutually repressive module that could coordinate the Pi and Fe signaling and homeostasis [81]. However, the underlying mechanism of Pi-Fe signaling interactions centered on PHR is still much unknown and needs to be further explored. At the end of the plant growth cycle, Fe is stored by chelating with Pi mainly in the seed vacuoles, which seems to limit Fe absorption in the intestine from the perspective of the human diet [82]. Currently, the understanding of the Pi-Fe interaction and the underlying specific mechanism remains limited, for example, Pi-Fe signaling interactions centered on PHRs in rice. In conclusion, future studies need to focus on how the Fe accumulation is influenced by other nutrients and the molecular basis for coordinating the mechanisms of interaction and homeostasis, which will help to identify new members involved in the Fe uptake and transport and improve the Fe accumulation in crops.

## 5. Strengthening Route of Micronutrient Fe Nutrition in Rice Grains

One grain-nutrient strengthening strategy is to explore and study the increasing contents or bioavailability of grain micronutrient nutrition through genetic and agronomic pathways. In particular, strengthening micronutrient Fe nutrition in food crops can serve as a pathway of mitigation and/or provide a solution to worldwide Fe deficiency [3]. Currently, unprecedented progress in rice high-yield breeding has been achieved, which has regretfully led to the loss of certain excellent and ancient genetic traits. These traits are potentially useful regarding the goal of grain high nutrition, such as in breeding and improving Fe-enriched rice with diversification resources and genetic information. Over the past few decades, studies based on the molecular mechanism of Fe homeostasis in plants have also explored potential or effective strategies for grain Fe nutritional strengthening in rice. The current strategies include three reinforcing methods, traditional breeding methods, transgenic biotechnology and agronomic optimization, which are acceptable for crop grain nutrition strengthening [83].

### 5.1. Strengthening Grain Fe Nutrition through Conventional Breeding Methods

For centuries, desirable food crop traits have been chosen to be bred with desirable high-yield targets. Due to the complex and strict network regulation of grain Fe and other elements, the study of crop grain Fe biological reinforcement has progressed slowly [84]. Traditional breeding relies on the diversity of Fe-enriched phenotype genetic traits in crops as well as the development of genetic markers, but rice varieties with Fe-enriched characteristics are often old, endemic, and/or wild species, which have been gradually ignored and lost due to their low production [85]. The modern breeding focus has emphasized the infiltration of special ideal traits in wild breeds into modern crops [86].

Genomic-wide association analysis (GWAS) and quantitative trait locus localization (QTL) that is associated with grain nutritional quality can be specifically identified and localized in the crop genome. Among them, GWAS was able to assess the impact of different single-nucleotide polymorphisms (SNPs) in populations to identify their important roles and polymorphisms for specific genes, especially to facilitate individual gene identification for crop high-Fe grain targets. GWAS analysis of 144 multiparent advanced-generation reciprocal line rice has already identified certain Fe-enriched and steady-state genes, including the NA synthesis gene *OsNAS3* and the *vacuolar iron transporter* (*VIT*) family gene *OsVIT1* [87]. In addition, QTLs are also a powerful tool to study polygenic quantitative traits. Certainly, several QTLs that are associated with grain micronutrient enrichment, including Fe and Zn, have been identified and localized in the rice genome using molecular markers [88,89,90]. Anuradha et al. (2012) identified 14 QTLs that are associated with the Fe and Zn concentration from a hybrid population, as well as 12 candidates for Fe and Zn homeostasis, such as *OsYSL1* and *OsMTP1* [91]. All of the different crop population sizes and types, different experimental designs or statistical methods and annual environments greatly affect the accuracy of QTL information; therefore, it is not possible to correspond and fully unified these results with the results of numerous studies. To elucidate specific genetic traits, it is necessary to integrate the QTL information data obtained from multiple experiments and project the date into the common atlas [92]. Due to the lack of corresponding genetic markers in the genome, QTLs generally correspond to large chromosomal regions. Combined GWAS and QTL analyses can help to refine chromosomal regions and identify specific genes.

### 5.2. Agronomic Optimization to Improve Rice Grain Fe Concentration and Bioavailability

Crop micronutrient reinforcement can be achieved by soil or leaf fertilization, particularly foliar fertilizer, which is recognized as a short-term solution to improve the utilization rate of fertilizer nutrients and to improve the nutritional status of crops. Genetically, crop engineered nutritional reinforcements may be more cost-effective in the long term, and current agronomic measures are more enforceable and are very effective in improving the mineral content of grain crops.

Mineral-enriched crop cultivation and planting are limited by the effective micronutrient quality of cultivated land. The effective Fe absorption and utilization from soil are also determined by the pH value and solubility of cultivated land [93]. For example, Fe generally exists as Fe^3+^ under dry land and oxidation conditions, maintaining and forming an insoluble Fe oxide/hydroxide form. In contrast, Fe usually exists as Fe^2+^ in anaerobic flooding conditions with better solubility and can more easily be absorbed and used. Therefore, soil fertilization that is based on reasonable Fe-source conditions is also an important factor to improve the Fe utilization rate in rice plants. Fageria et al. (2002) found that synthetic chelates are effective for applying Fe fertilizer in soil but are limited due to their high cost [94]. The FeSO_4_ solution is commonly applied, but it was noted that the inorganic solution applied was readily fixed into an insoluble form that was immersed in soil with low effects [95]. Nonetheless, soil/leaf Fe fertilization must be used as a complementary practice to improve crop Fe accumulation. For other trace elements, it has been found that leaf fertilization with Zn is very effective with increasing grain Zn under different soil conditions due to leaf Zn remobilization into grains during the rice grouting period [96]. The present study found that H^+^ can be released by the rational application of organic fertilizer, reducing the pH value of soil and thus increasing the soil Fe solubility and availability. Compared to rhizosphere fertilization, the fertilization of leaf micro fertilizer has higher bioavailability and greater advantage in protecting the environment. Wang et al. (2020) applied the appropriate concentration of leaf Fe fertilizer, which not only improved rice yield and grain Fe concentration but also reduced the unavailable Fe-phytic acid level and grain Cd concentration by 29% [66]. In summary, rice grain Fe concentration is affected by multiple factors, and an effective, safe, and sustainable development strategy should be considered and carried out in combination with these factors.

In agricultural production, important agronomic methods have been widely explored to optimize the absorption of leaf fertilizer application and bioactivity for yield and quality improvements [97]. In contrast to the application of soil fertilizer, utilizing leaf fertilizer can directly and effectively increases crop micronutrient contents within a short term. Similar to the soil fertilization stage, spraying leaves with micro fertilizer during spike differentiation is more effective at improving the micronutrient content in rice grain. At the flowering stage, a zinc application of 0.5% *w*/*v* ZnSO_4_ can significantly improve the grain-zinc content in rice with a grain-zinc increase to two-fold [98]. Leaf spraying helps to avoid low Fe utilization in the soil, and considering the optimal spraying time is very important to effectively improve the grain Fe level. Some studies have compared four different forms of foliar Fe fertilizer, including FeSO_4_, EDTA-FeNa, DTPA-Fe, and HEDTA-Fe, and the studies showed that all of these forms could improve the grain Fe concentration and reduce the phytic acid content in rice endosperm, thus enhancing Fe bioavailability in rice grain. The foliar fertilizer of DTPA-Fe and FeSO_4_ was significantly better than that of the other two fertilizers [99]. It has also been shown that spraying leaves with Fe-amino acid (Fe-AA) chelate increases the rice grain Fe concentration, especially with the addition of 1% (*w*/*v*) Fe-NA, which resulted in a 32.5% increase [100]. Our group study has carried out in rice plants by spraying with different concentrations of ZnSO_4_/FeSO_4_ during the tillering and booting stages. The results have shown the spraying treatments should be successfully able to improve the photosynthesis efficiency and to enhance the grain pigment accumulation in the coloured rice [101,102]. Notably, foliar spraying with FeSO_4_ can also increase the grain Fe concentration by 2.5~1.3-fold in different rice varieties (unpublished data). Compared with soil fertilization, the application of microfertilizer has the advantage of economic, effective and sustainable development.

Nanoparticles (NPs) have a great potential in nanofertilizer applications involving agronomic optimization to improve the efficiency of agrochemicals to a greater extent [103,104]. In production, the application of Fe-based nanoparticles can improve crop growth and ferritin accumulation. Li et al. (2021) found that low doses of zero-valent iron (ZVI)-/Fe_3_O_4_-NPs can be used as an alternative to conventional Fe fertilizer to ensure normal growth under Fe-deficient conditions [105]. The latest study has shown that the addition and application of Fe-nanoparticle fertilizer reduced the toxic accumulation of heavy metal elements, such as Cd and As, in rice plants [106,107]. In addition, some negative effects of Fe-based nanoparticles have been reported. For example, exogenous ZVI-NPs at a concentration of 500 mg L^−1^ can induce excess ROS accumulation, causing DNA damage and a reduction in the mitotic index in plant cells [108]. Nanofertilizers are controversial in such applications. Therefore, more research is needed to fully understand the impact of Fe-based nanomaterials before they can be safely applied in agricultural production.

### 5.3. Genetic Engineering Technology Improves Fe Capacity in Rice Grain

There is a limited range of genetic variation in the endospermic Fe levels in rice germplasms. Traditional breeding focuses on the excavation and breeding of Fe-rich rice resources. At present, nutritional grain Fe reinforcement has not yet been achieved in conventional staple food varieties. Using Fe absorption, transportation, and storage genetic engineering, modern breeding offers a complementary method to explore and create Fe-rich rice resources. Based on the progress in Fe absorption, transport, storage, regulation, and homeostasis, the current transgenic strategies and pathways for Fe bioreinforcement in crops are generalized [109,110,111]. Among the strategies, the more effective genetic bioreinforcement pathway includes (i) enhancing the absorption of soil-derived micronutrients, (ii) enhancing the transport of micronutrients into grains, (iii) increasing the accumulation of endosperm tissue-specific minerals, (iv) reducing the accumulation of antinutrient factors in grains, and (v) improving grain mineral bioavailability [84,112].

In the study of the Fe absorption mechanism, many of the genes involved have been identified in rice, among which target genes have also been used for rice genetic bioreinforcement. For Fe absorption in rice, overexpressing the transporter gene *OsIRT1* induced a 1.7- and 1.1-fold increase in leaf and grain Fe concentrations, respectively [52]. It seems that it may be necessary for rice grain Fe reinforcement to both enhance its absorption and transport capacity, as well as strengthen the tissue-specific storage capacity of Fe. By transgenic technology, chloroplast-derived ferritin can be expressed in rice endosperm, and it is an efficient way to enhance the Fe storage capacity of rice endosperm. The tissue-specific promoter of globulin and glutelin drives ferritin expression in rice endosperm with a grain Fe increase of 2.0 to 3.7 times [46,56,113,114,115].

To improve Fe uptake and transport capacity in overall plants, certain strategies have been explored, including upregulating the induced Fe chelator synthesis pathway and regulating and optimizing inter-/intracellular transport and storage. The overexpression of *NAS* constitutive and PS synthesis genes in rice plants promotes the upregulation of Fe chelator synthesis NA and PS, which increases the grain Fe concentration in polished rice by two to four times [25,26,59]. The constitutive expression of the *HvYS1* barley gene in rice under the controlled maize *ubiquitin 1* (*ubi-1*) promoter moderately promotes Fe transport from roots to seeds with an increase in grain Fe accumulation [116]. The *iron deficiency-specific clone 3* (*IDS3*) gene derived from barley (*Hordeum vulgare* L.) can be expressed in rice, regulating the upregulation of Fe transport pathway genes and then increasing the grain Fe level by 1.4-fold in polished rice [117]. Rice plants with overexpression of *OsYSL15* and *OsYSL2* that is controlled by the promoter of the actin or sucrose transporter gene can have increased *YSL* expression level in various tissues, prompting a 1.2- to 4.0-fold increase in grain Fe concentration [27,30]. Senoura et al. (2017) found that *OsYSL9* expression is depressed in rice plants through RNA interference (RNAi), which also impairs Fe transport from the endosperm to embryo, increasing the embryonic Fe concentration [37]. Thus, *OsYSL9* is a good target gene for bioreinforcement. In rice, regulating Fe intracellular transport by decreasing *OsVIT1* or *OsVIT2* expression can promote inter-tissue Fe transport from flag leaves to grains, eventually increasing the endosperm Fe concentration [118,119,120]. Fe accumulation in polished rice can be altered by regulating the stem vacuolar mugineic acid transporter gene *OsVMT*, which is a vacuole membrane protein responsible for DMA transport into vacuoles. *OsVMT* knockdown can increase DMA in the cytosol to dissolve and chelate more Fe, resulting in a grain Fe transport and accumulation increase by 1.0- to 2.1-fold compared to that of the wild-type [121].

In rice plants, Fe homeostasis regulators also indirectly influence the network of Fe transport and accumulation. For example, the transcription factor Fe-related transcription factor 2 (OsIRO2) is involved in regulating the biosynthesis of Fe chelate, MA, and the Fe-MA transport process. In calcium-based Fe-deficient soil, rice plants constitutively expressing *OsIRO2* can still grow normally with a threefold increase in grain Fe concentration [122]. In addition, it has been reported that knockdown of negative regulator, zinc finger protein 1 and zinc finger protein 2 (OsHRZ1/OsHRZ2), can significantly promote grain Fe accumulation in rice [123].

Compared to the single-gene strategy, a gene-combination strategy related to effective Fe transport and storage has achieved a more significant endosperm Fe increase in rice. The cooperative overexpression of *AtIRT1*, *AtNAS1*, and *PvFER* in rice plants can increase Fe concentration not only in brown rice but also in polished rice, with endosperm Fe reaching 10.46 mg kg^−1^, which seems more efficient than transgenic lines with a single gene insertion [124]. Similarly, the endosperm Fe concentration increased up to 55 mg kg^−1^ by the synergistic overexpression of *OsNAS1* and *HvNAAT* in *japonica* rice [125]. Aung et al. (2013) overexpressed *HvNAS1*, *OsYSL2* and soybean *FER* in rice varieties widely cultivated in Myanmar and Japan, and their Fe content in polished rice increased by 3.4 to 6 times, respectively [126].

Furthermore, genome editing methods have great potential for the bioreinforcement of micronutrients in rice. The latest genome editing tool is clustered regularly interspaced short palindromic repeats (CRISPR) for precise modifications, which provides the possibility of precise targeting genes or genomic regions. Knockdown of the *OsVIT2* gene based on the CRISPR method can increase the Fe amount in grains [119]. The development of RNAi-mediated gene silencing technology has led to studies being conducted to reduce *inositol 1,3,4,5,6-pentakisphosphate 2-kinase* (*IPK1*) gene expression by RNAi [127]. In conclusion, important progress has been made in studies on implementing Fe bioreinforcement using genetic engineering, as summarized in (Table 1). As science and technology constantly evolve, more methods can be integrated into breeding and genetic engineering projects that target complex micronutrient biological reinforcement.

## 6. Suggestions for Further Research

Human iron deficiency is a widespread and increasingly serious health problem, and the biological strengthening of Fe in staple food is an effective way to address and alleviate this problem. Fe-enriched varieties can be developed and promoted as functional foods or alternative staple foods to ensure that people have adequate daily sources of nutritional Fe, particularly in developing countries that have rice as a staple food. According to the progress seen in the above research, the following suggestions are developed for future research directions and strategies for Fe biological strengthening in rice.

First, traditional resources can be used to develop a variety of Fe-reinforced rice varieties to meet the needs of different agricultural ecosystems. Although the range of genetic variation in rice germplasm is not sufficient to achieve its Fe-rich targets, there is a degree of variation among different varieties that affects grain Fe concentrations. Originally, the Fe(III)-DMA transporter encoded by *YSL18* was expressed in rice phloem and had a higher expression of *YSL18* in flag leaves from Fe-enriched varieties compared to that of other ordinary varieties [35,128]. This suggests that the speed-limiting steps for increasing grain Fe concentration are also variety-specific. This discrepancy may manifest as different effects of certain biological reinforcement strategies under a genomic background. For different rice varieties, a restriction-factor analysis for their grain Fe levels, which are especially common as targets for biological reinforcement within geographic regions, will be useful to identify genetic germplasms suitable for biological reinforcement. Bioreinforcement strategies must also be tested in a growing number of common varieties that are widely planted worldwide to enter the application stage. Genetic variation is significant not only for rice Fe concentration but also to avoid the accumulation of toxic metals. Currently, although it is unclear whether Cd content in Fe-bioreinforced lines is affected, the main quantitative trait loci for the Cd content and the corresponding genomic markers have been identified. This knowledge would facilitate procedure screening against a Fe-rich variety background to reduce the risk of toxic metal accumulation [129,130]. The absorption of toxic metals, including As, Cd, Hg, and Pb, is known to be negatively correlated with Fe plaque formation on the root surface, which is caused by root oxygenation and differences with different genotypes in rice [131,132]. Therefore, to ensure that the roots of Fe-bioreinforced lines can still form enough Fe plaque to block toxic metal absorption, screening iron-fortified background cultivars from their root oxygenation capacity can be considered.

Genetic engineering is a more suitable biological reinforcement strategy to quickly break through the bottleneck or limit of improving grain-micronutrients in rice. For more than a decade, transgenic or gene-editing methods have been beneficial for rice nutrition biology reinforcement, including the efficient expression of Fe absorption, transport and accumulation of related genes, reduced competitive gene expression to improve Fe in rice, and improved ferritin bioutilization as well as synchronous multigene efficiency strategies, endosperm-specific reinforcement strategies, and collaborative micronutrient accumulation angles.

Although the molecular mechanisms of Fe homeostasis in rice have been gradually elucidated, the mechanistic complexity is not fully understood, and further molecular mechanisms will also contribute to the diversification of Fe biological reinforcement strategies. The type of rice that people eat is mostly polished refined rice, thus gene Fe biological reinforcement studies should consider the specific accumulation of Fe in tissue and especially the improvement of endosperm Fe concentration in order to meet the daily dietary needs. Among these priorities, the expression of genes involved in the long-distance transport of Fe and Fe storage, such as *YSL*, *NAS* and *FER*, are key to increasing endosperm Fe. The Fe bioavailability of rice is also one of the priorities of biological enhancement. The current in vitro assays based on Caco-2 cells and animal feeding experiments show that constitutive *NAS* expression or endosperm-specific *FER* is susceptible to intestinal absorption, but the study of Fe bioavailability is still in its primary stage. Therefore, future research on whether Fe-bioreinforced rice can improve the health of Fe-deficient people is the main focus, and in addition to the above strategies, strategies to improve bioavailability still need to be studied.

Second, the relationship between nutritional Fe-enriched characteristics, planting environments and genetic backgrounds should be emphasized, and cultivation-regulation means should be used to improve grain Fe quantities to accomplish nutritional biological strengthening. Environmental factors, such as Fe absorption and transportation mechanisms, water management, and soil pH, the application amounts of nitrogen and phosphorus fertilizer, and even changes in atmospheric conditions, will affect Fe enrichment or reinforcement in rice. Water management controls the solubility of Fe from soil by affecting its redox potential, thus the rice grain effective Fe capacity is also affected. Therefore, different water management protocols can be formulated based on the variation in Fe absorption capacity of Fe-rich or Fe-reinforced transgenic rice lines. According to agronomic fertilization, the main problem is environmental pollution. Although most micronutrient fertilizers are not easy to leach, they combine firmly with the soil very easily, and the continuous use of trace nutrient fertilizer can lead to mineral accumulation, which can lead to toxic effects. Therefore, it is necessary to design and optimize fertilization measures. Among the measures, leaf fertilization is more environmentally friendly than soil fertilization is, which causes less pollution, but the cost of leaf fertilization is relatively high. Future research on foliar fertilizer needs to emphasize multiform and low-cost efficient fertilizer, and it can also consider the development of new nanoparticles combined with fertilizer to further improve the utilization efficiency.

## 7. Concluding Remarks

Over the past few decades, different Fe-trophic bioreinforcement strategies have been explored in rice, and some of these strategies have also been demonstrated to effectively improve grain Fe levels. Nevertheless, there are still challenges in food safety and nutritional effects. Therefore, both plant scientists and nutritionists should be involved in the study of nutritional biological reinforcement strategies, the promotion and application of food crops, nutritional utilization, and food safety.

## Figures and Tables

**Table 1:** Biofortification of Fe in rice using genetic engineering techniques.

Gene	Method	Tissue	Strengthen Fe Level	Reference
**Absorption and transport**				
*OsIRT1*	overexpression	brown rice	1.1-fold	[52]
*OsYSL15*	overexpression	brown rice	1.2-fold	[27]
**Long-distance transport**				
*HvIDS3*	transgenic plant	polished rice	1.4-fold	[117]
*OsYSL*2	overexpression	polished rice	4.0-fold	[30]
*ubi-1* + *HvYS1*	constitutive expression	endosperm	1.9-fold	[116]
*OsYSL9*	RNAi	embryo	~3.0-fold	[37]
**Storage protein**				
*Py*FER	transgene	brown rice	2.0-fold	[113]
*GmFER*	transgene	brown/polished rice	3.0~3.7-fold	[56]
*SoyFER H*1	transgene	brown rice	1.3-fold	[115]
*O*sFER2	overexpression	brown rice	2.1-fold	[114]
*GmFER H*1	transgene	polished rice	3.4-fold	[46]
**Vacuole-related transport/storage**				
*OsVIT1/OsVIT2*	mutation	brown rice	~1.5-fold	[118]
*OsVIT2*	T-DNA insert	brown/polished rice	>1.5-fold	[109,120]
*OsVMT*	knockout	polished rice	1.8~2.1-fold	[121]
**Chelate synthase and combination strategy**				
*OsNAS1*/*OsNAS*2/*OsNAS*3	overexpression	brown rice	2.0~4.0-fold	[25,26]
*HvNAS1, OsYSL2, SoyFER*	overexpression	polished rice	3.4-fold	[126]
*AtIRT1,* AtNAS1, PvFER	overexpression	endosperm	4.2-fold	[124]
*OsNAS1, HvNAAT*	overexpression	embryo/endosperm	1.3~2.9-fold	[125]
**Transcription factor**				
*OsIRO2*	overexpression	brown rice	3.0-fold	[122]
*OsHRZ1/OsHRZ2*	knockout	brown/polished rice	2.9~3.8-fold	[123]

## Data Availability

All data are presented in the review manuscript in the form of tables and figures.
